# A systematic study of microdosing psychedelics

**DOI:** 10.1371/journal.pone.0211023

**Published:** 2019-02-06

**Authors:** Vince Polito, Richard J. Stevenson

**Affiliations:** 1 Department of Cognitive Science, Macquarie University, Sydney, Australia; 2 Department of Psychology, Macquarie University, Sydney, Australia; King's College London, UNITED KINGDOM

## Abstract

The phenomenon of ‘microdosing’, that is, regular ingestion of very small quantities of psychedelic substances, has seen a rapid explosion of popularity in recent years. Individuals who microdose report minimal acute effects from these substances yet claim a range of long-term general health and wellbeing benefits. There have been no published empirical studies of microdosing and the current legal and bureaucratic climate makes direct empirical investigation of the effects of psychedelics difficult. In Study One we conducted a systematic, observational investigation of individuals who microdose. We tracked the experiences of 98 microdosing participants, who provided daily ratings of psychological functioning over a six week period. 63 of these additionally completed a battery of psychometric measures tapping mood, attention, wellbeing, mystical experiences, personality, creativity, and sense of agency, at baseline and at completion of the study. Analyses of daily ratings revealed a general increase in reported psychological functioning across all measures on dosing days but limited evidence of residual effects on following days. Analyses of pre and post study measures revealed reductions in reported levels of depression and stress; lower levels of distractibility; increased absorption; and increased neuroticism. To better understand these findings, in Study Two we investigated pre-existing beliefs and expectations about the effects of microdosing in a sample of 263 naïve and experienced microdosers, so as to gauge expectancy bias. All participants believed that microdosing would have large and wide-ranging benefits in contrast to the limited outcomes reported by actual microdosers. Notably, the effects *believed* most likely to change were unrelated to the *observed* pattern of reported outcomes. The current results suggest that dose controlled empirical research on the impacts of microdosing on mental health and attentional capabilities are needed.

## Introduction

Microdosing refers to the practice of ingesting a very low dose of a psychedelic substance [1]. There has been little peer-reviewed research on microdosing but there are numerous blogs and online communities that discuss the practice, with detailed guides to methods and anecdotal reports of outcomes (e.g., www.microdosing.com; www.reddit.com/microdosing/wiki). Typical doses can be as small as one twentieth of a typical recreational dose, sometimes even less [2]. So, for example, a microdose of lysergic acid diethylamide (LSD) might be 6–25 micrograms, or a microdose of psilocybin might be .1 to .5 grams of dried mushrooms [3]. People microdose using a wide range of different substances, although LSD and psilocybin are the most commonly discussed in online forums [4].

Psychedelics have typically been associated with marked alterations in cognition, affect, perception, and neurophysiology [5]. Individuals who have taken psychedelics typically describe pronounced changes in visual and auditory perception, accompanied by vivid imaginative experiences and intense emotions. This is not the case with microdosing. Microdosing is frequently described as involving a ‘sub-threshold’ dose [6]. That is, individuals aim to identify a dose at which they do not feel ‘high’. In other words, when microdosing there are only minimal identifiable acute drug effects.

People follow a variety of different schedules when microdosing, sometimes taking a dose each day but much more frequently interspersing dosing days with rest days. One common schedule is to microdose every three days [7]. The idea behind this regimen is that there may be a residual effect from each microdose that lasts one to two days afterwards. Most popular press stories on microdosing have mentioned this three day cycle [8,9].

Despite the reported lack of acute effects of microdosing, proponents claim a wide variety of psychological and social benefits from regular microdosing, including increases in vitality, creativity, productivity, social ability, focus, analytic thinking, positive mood, memory, mindfulness and general wellbeing [10]. Microdosing is thus a curious phenomenon: on the one hand advocates deny experiencing the alterations in consciousness that characterise typical doses, yet claim significant psychological benefits from regular use.

The earliest occurrence of microdosing is unknown. Anthropological reports indicate that many traditional cultures incorporated use of psychedelic plants such as peyote, morning glory seeds and psilocybin containing mushrooms into many aspects of daily life [11]. These substances were used as a catalyst for ritual religious experience [12], but also used at lower doses as an aphrodisiac, to reduce hunger, inspire courage, nullify pain, and to treat ailments such as gout and syphilis [13]. These uses highlight that although psychedelics are now commonly associated with marked alterations in consciousness, they also have also been used historically at low doses for therapeutic benefits and functional enhancement.

The modern practice of microdosing is quite a recent phenomenon. Albert Hofman, the discover of LSD, mentioned the use of very low doses of LSD (25 micrograms) in passing during in a 1976 interview [14] but we have not been able to identify any other records from Dr Hofman or his contemporaries describing microdosing. Stanislav Grof developed psycholytic psychotherapy [15] as a form of psychedelic assisted therapy that involved small amounts of LSD, but the lower range for doses was over 100 micrograms—considerably higher than contemporary microdosing. There was no formal research on microdosing prior to the prohibition of psychedelic research in 1966.

The current popularity of microdosing can be traced back to a book, *The Psychedelic Explorers Guide* by James Fadiman [1]. This was the first publication to describe microdosing in detail. Fadiman outlined the purported benefits of regular microdosing, with a recommendation to follow a three-day cycle, and guidelines for appropriate doses. This publication also contained a collection of case reports from individuals about their microdosing experiences, emphasising positive improvements in creativity, focus, affect, and relationships. In the years following this publication, a number of news articles appeared reporting on the growing interest in microdosing. The first major publication to report on the phenomenon was *Rolling Stone* [16]. This article triggered considerable popular media interest in microdosing psychedelics that has led to over 1200 news articles on the topic since that time. Many stories in the popular press have focused on microdosing as tool for increased productivity, particularly for people working in the technology sector [17–19]. This sudden high level of almost exclusively positive news coverage has been accompanied by the emergence of multiple communities of microdosers on social media. For example, a forum on microdosing on reddit.com has over 24,000 members who report on their experiences and compare notes on methodologies, outcomes and protocols. The comprehensive news coverage and active online communities of microdosers have led to a situation where large numbers of individuals are experimenting with microdosing, with the expectation that this practice leads to substantial psychological and wellbeing benefits.

To date there are four scientific articles on microdosing. Three of these indicate potential benefits from microdosing. First, Johnstad [20] conducted a series of interviews with microdosers, who reported generally positive outcomes, including improved mood, energy levels and cognition. Second, in an open label study, Prochazkova et al. [21] found that microdosing led to increases in convergent and divergent thinking–common indicators of creativity. Third, a large cross sectional study found that microdosers reported reduced levels of negative attitudes and emotions, and increased wisdom, open-mindedness and creativity, relative to people who had never microdosed [22]. Fourth, the most scientifically rigorous study to date, was a double blind placebo controlled study by Yanakieva et al. [23]. This study showed changes in time perception following microdosing, but did not investigate variables related to health or wellbeing.

In addition to this formal research, microdosers’ expectations are likely based on informal case reports, anecdotes, unpublished studies, and online publications [3,24–26]. Although microdosing is understudied, its sudden popular interest has occurred within a context of growing scientific attention on the effects of psychedelics taken at higher doses [27]. After a long period of minimal research with psychedelic compounds as a result of government prohibitions, an increasing number of research teams have in recent years reported compelling findings suggesting both improved psychological functioning [28] and potential therapeutic benefits [29] associated with a range of psychedelic substances.

When administered to healthy individuals in a supportive setting, both psilocybin [30–33] and LSD [34], have been shown to elicit mystical-type experiences that are characterised as highly meaningful and transformative by participants. In the case of psilocybin, Griffiths et al. [31] reported persisting self and observer rated positive effects on attitudes, mood and behaviour 14 months after ingestion, with 58% of participants reporting that their psilocybin experience was among the five most personally meaningful experiences of their lives. Psilocybin [35] and LSD [36] have also both been shown to bias emotional processing toward positive information and to attenuate responses to fearful stimuli. In addition psilocybin has been shown to increase emotional empathy [37], whereas LSD has been shown to increase feelings of well-being, closeness to others and trust [38]; increase emotional response and personal meaningfulness to music [39,40]; and increase suggestibility [41].

In more clinically oriented research, both psilocybin and LSD have shown promise as treatments for end of life anxiety. In a double blind, randomised crossover trial of psilocybin assisted psychotherapy as a treatment of anxiety and depression in terminal cancer patients, Griffiths et al. [42] found remission in both depressive and anxious symptoms for over 60% of patients at 6 month follow up. A similar trial of LSD assisted psychotherapy by Gasser, Kirchner and Passie [43] found significant reductions in state and trait anxiety that were maintained at 12-month follow up. Psilocybin has also been shown to reduce the symptoms of treatment resistant depression [44], and to dramatically reduce consumption levels when trialled as a treatment for tobacco addition [45,46], and alcohol dependence [47].

As a result of this increased research activity, researchers are developing a clearer picture of the psychopharmacological mechanisms that underlie psychedelic substances. A comprehensive review of the mechanisms of action of psychedelics is provided by Nichols [5]. Briefly, it is well established that classic serotonergic psychedelics such as psilocybin, dimenthyltryptamine (DMT), mescaline and LSD act (at least partially) through an agonistic effect on 5-HT_2A_ receptors throughout the brain [48], and recent work has suggested that long term use of psychedelics may lead to structural changes in the posterior and anterior cingulate cortices [49]. Recent research has also indicated that psychedelics lead to reduced activity in the default mode network, a network of brain regions thought to support general background activity associated with the functions of normal waking consciousness, such as metacognition, social attributions and self reflection [50]. This reduction in typical neural activity is accompanied with an increase in connectivity between brain regions that usually function relatively independently [51].

New findings on the effects of higher dose psychedelics are being published at a rapid rate, and overall the emerging research suggests that these substances may have beneficial impacts across a range of psychological, cognitive, affective, and psychosocial domains. These findings have informed the cultural narrative around microdosing and the broad scope of constructs that appear to be influenced by psychedelics taken at higher doses is consistent with the diverse range of effects described by proponents of microdosing. Furthermore, research into the role of psychedelics in the brain provides at least a plausible account of the potential neural mechanisms of microdosing.

There has been no specific research into the safety of microdosing, however research with higher doses of psychedelics suggests that these substances are relatively safe [52]. Individuals do sometimes have disturbing experiences on psychedelics, including negative emotions, perceptual disturbances, and even psychotic symptoms, and these effects can have a persisting negative impact for some people [53,54]. In general, however, psychedelics are not addictive [55], and large scale population studies have not found any association between use of psychedelics and negative mental health outcomes [56]. Longitudinal research even suggests that lifetime use of psychedelics may be associated with lower levels of psychological distress [57] and reduced suicidality [58]. A number of studies have ranked the comparative risks of different types of drugs for both the user and broader society; these studies have consistently found that psychedelics are among the least harmful substances, with far less personal and societal risks than legal drugs such as alcohol and tobacco [59,60]. The doses involved in microdosing are considerably smaller than typical doses and so it may seem reasonable to assume that any risks would be diminished, but it is worth noting that psychedelics are usually taken relatively infrequently, even by enthusiasts. It is possible that chronic low-dose exposure to psychedelics, as occurs in microdosing, may involve unknown risks [61].

The motivation for the current study was to attempt to resolve some of the unknown questions around microdosing. There is currently considerable popular interest in this practice with indications that a great many individuals are experimenting with regular low doses of psychedelics [9]. Online discourse on microdosing presents a wide range of potential positive benefits. The prominence of these positive claims may lead individuals to develop expectations about what their own experience of microdosing will be like, and these expectancies themselves may influence participants’ reports. The potential role of expectancy effects may be amplified by two factors. First, most individuals who become curious about microdosing would likely need to go to some effort (and some risk) to source and prepare psychedelic substances in order to begin experimenting. It is reasonable to suppose that this effort would lead to a sense of investment in the expected outcome, and may bias individuals to perceive the effects they are expecting. Second, as described above, the immediate effect of each microdose is only a very minimal (“subthreshold”) alteration in consciousness. Microdosers may interpret these subtle and ambiguous effects in line with their expectations. We explore the role of expectancy or placebo effects in Study Two.

On the other hand, there are plausible neurobiological mechanisms that may account for microdosing effects, and a growing body of research suggesting a range of reliable positive benefits from higher doses of psychedelics [62]. There have been no published empirical studies of microdosing and it is unclear how accurate anecdotal reports of benefits are. The current legal and bureaucratic climate makes direct empirical investigation of the effects of psychedelics difficult and the approval process for experimental work is prohibitively lengthy. With this background, we designed a study that could be completed relatively rapidly, as an initial exploratory investigation into the effects of microdosing.

We aimed to carefully track a cohort of healthy individuals who microdose in a systematic, observational study to see if measures taken using well validated psychometric assessments would match anecdotal claims regarding the effects of microdosing. Although individuals microdose with a wide range of substances we restricted our analysis to a group of substances with similar psychopharmacology: serotonergic psychedelics [63,64]. This subclass of substances (which includes LSD, psilocybin, mescaline and number of synthetic ‘research chemicals’) all act upon 5-HT receptor sites.

We identified psychological variables that may be affected by microdosing in three ways: by reviewing descriptions of microdosing effects in online forums and media reports; by reference to scientific reports of the effects of higher doses of psychedelics [30,44,65–67]; and in consultation with James Fadiman (personal communication, February 20, 2016). Nine domains were selected for investigation: mood, attention, wellbeing, mindfulness, mystical experiences, personality, absorption, creativity, and sense of agency.

## Study One

In Study One we tracked the experiences of microdosers over a period of six weeks. This time period was selected to provide enough time for microdosers to complete multiple dose-rest cycles, and to allow a reasonable amount of time to elapse between baseline and post-study measures. We tracked participants in two ways: through daily emails asking for brief ratings of psychological functioning, and through comprehensive questionnaire batteries at baseline and at completion of the study.

## Method

Supplementary materials and data from this project are available at on the Open Science Framework at osf.io/6xfm8/

### Participants

Participants were recruited through posts on online communities of microdosers. A recruitment notice was posted on the websites reddit.com/r/microdosing, bluelight.org, and in psychedelic discussion groups on facebook.com. The study was open from April 2016 to April 2017.

1181 participants clicked through to the study webpage. Of these, 251 (21.25%) participants completed the baseline questionnaire. We eliminated duplicate responses (*n* = 4), and also excluded participants who did not report any microdosing during the study period (*n* = 149). 98 participants sent at least one daily report of microdosing with a serotonergic psychedelic and were included in the analysis of daily ratings. 63 of these participants completed the post-study questionnaire and were included in the long term analysis of pre and post study measures. Of these 63, 14 (22.2%) participants were female, 49 (77.8%) were male. In order to maximise anonymity participants nominated age ranges rather than divulging exact ages. 13 (20.6%) participants were aged 18–25, 31 (49.2%) were aged 26–35, 11 (17.5%) were aged 36–45, 7 (11.1%) were aged 46–55, and 1 (1.6%) was aged 56+. Participants were mainly from Australia (29.0%), USA (17.6%) and Canada (12.9%). The sample was highly educated with 71.4% participants having completed postgraduate education. 19.0% participants were students and 76.2% were working full time or part time.

Based on responses taken at baseline, this sample was experienced with psychedelics but reported relatively moderate experience with other drugs. Specifically, 6 participants (9.5%) reported using psychedelics more than once a week, 14 (22.2%) used psychedelics a few times a month, 33 (52.4%) a few times a year, and 10 (15.9%) had no prior experience of psychedelics. We also asked participants about their use of 3,4-methylenedioxymethamphetamine (MDMA), methamphetamine, opiates and other similar drugs (excluding cannabis). 2 participants (3.2%) reported using other drugs more than once a week, 5 (7.9%) a few times a month, 30 (47.6%) a few times a year, and 26 (41.3%) reported no previous experience with other drugs.

### Procedure

Upon accessing the study webpage participants were presented with an overview of the study requirements and safety information (see supplementary material at osf.io/6xfm8/). Participants were informed that in order to take part they must be aware of microdosing, regardless of whether or not they had actually ever microdosed. Participants were asked not to take part if they had any history or current diagnosis of primary psychotic disorder, mood disorder, anxiety disorder, dissociative disorder or substance disorder. All interactions with participants were conducted through automated emails and online Qualtrics surveys. Participants were provided with instructions on how to create an anonymous email account and encouraged to use this to sign up to the study. When first signing up, participants completed a baseline battery of measures investigating mood, attention, wellbeing, mindfulness, mystical experiences, personality, absorption, creativity, and sense of agency. At the completion of the study (i.e., after 6 weeks) participants repeated this battery of measures. At post-study participants also completed the Altered States of Consciousness Rating Scale [68] and the Persisting Effects Questionnaire [30]. These measures were not analysed in the current study, but for completeness summary statistics are provided as Table A in S1 File. The baseline and post-study batteries each took approximately 50 minutes to complete. Additionally, participants received a daily email each day for a period of 42 days with a link to complete a brief series of daily ratings about their experiences. Daily ratings took less than 5 minutes to complete. Participants were not offered any incentive to take part in the research. Ethics approval for this study was provided by Macquarie University Ethics Committee and electronic consent was provided by each participant.

### Materials

#### Daily ratings

Participants were invited to complete a brief rating scale each day during the study, adapted from Fadiman’s [2] protocol. Participants were first asked if they had microdosed on the previous day. If they had, they were asked to report the substance and dosage. Participants then gave a single rating for feelings of each of the following: *Connectedness*, *Contemplation*, *Creativity*, *Focus*, *Happiness*, *Productiveness*, and *Wellbeing* during the previous day (e.g. “How connected did you feel yesterday?”). Ratings were made on a 5-point Likert scale from ‘much less than average’ to ‘much more than average’. This scale was designed for brevity and took less than two minutes to complete.

#### Long term measures

Participants completed a comprehensive battery of questionnaires when first signing up for the study and then again at the conclusion of the study, after 6 weeks of microdosing. This battery investigated nine domains of psychological functioning. These are described below and summarised in Table 1.

**Table 1:** Psychological domains investigated in long term analyses.

Domain	Measure	Subscale	Target
Mental Health	Depression, Anxiety, Stress Scale (DASS) [69]	Depression	Severity of depression symptoms.
		Anxiety	Severity of anxious symptoms.
		Stress	Severity of stress symptoms.
Attention	Mind Wandering Questionnaire (MWQ) [70]	Mean MWQ score	Deviations in attention away from the task at hand.
Wellbeing	Quality of Life Inventory (QOLI) [71]	QOLI total score	Overall satisfaction with life.
Mindfulness	Mindful Attention Awareness Scale (MAAS) [72]	Mean MAAS score	Frequency of mindful, receptive awareness.
Mystical Experience	Hood Mysticism Scale (HMS) [73]	HMS total score	Degree of mystical experience.
Personality	M5P Personality Questionnaire [74]	Extraversion	Tendency to seek social company.
		Agreeableness	Tendency toward cooperation.
		Conscientiousness	Tendency to organized, dependable behavior.
		Neuroticism	Tendency to experience unpleasant emotions.
		Openness	Tendency to seek new experiences.
Absorption	Tellegen Absorption Scale (TAS) [75]	TAS total score	Disposition toward intense imaginative experiences.
Creativity	Creative Personality Scale (CPS) [76]	CPS total score	Overall creativity.
Sense of Agency	Sense of Agency Rating Scale (SOARS) [77]	Involuntariness	Feeling that events are externally generated.
		Effortlessness	Feeling of actions occurring spontaneously.

**Mental Health: Depression and Anxiety Stress Scale (DASS) [69]**. The DASS questionnaire measures the severity of depression, anxiety and stress symptoms. We used the 21-item version of the scale. Participants rated the frequency or severity of items such as “I tended to over-react to situations” over the previous week on a 4-point Likert-type scale (from “did not apply to me at all” to “applied to me very much”). We used all three subscale scores in our analyses: *Depression*, *Anxiety* and *Stress*.

**Attention: Mind Wandering Questionnaire (MWQ) [70]**. The MWQ is a five item, unidimensional scale that measures deviations in attention away from the task at hand. Participants rate their level of agreement on items such as “I have difficulty maintaining focus on simple or repetitive work” on a 6-point Likert-type scale (from “almost never” to “almost always”). We used the mean MWQ score in our analyses.

**Wellbeing: Quality of Life Inventory (QOLI) [71]**. The QOLI is a 32 item scale that measures life satisfaction across 16 dimensions. Participants rate the importance of each life dimension on a 3-point Likert-type scale (from “not Important” to “important”) and their level of satisfaction with each life dimension on a 6-point Likert-type scale (from “very dissatisfied” to “very satisfied”). Due to a coding error, scores were only recorded for 11 life dimensions: health, self-esteem, goals and values, money, work, play, learning, helping, love and friends. A modified total QOLI score was calculated by multiplying the importance and satisfaction ratings for each dimension and then summing these scores.

**Mindfulness: Mindful Attention Awareness Scale (MAAS) [72]**. The MAAS is a 15 item, unidimensional scale that measures the frequency of mindful, receptive awareness of the present moment. Participants rate their level of agreement with items such as “I do jobs or tasks automatically, without being aware of what I’m doing” on a 6-point Likert-type scale (from “almost always” to “almost never”). We used the mean MAAS score in our analyses.

**Mystical experiences: Hood Mysticism Scale (HMS) [73]**. The HMS is a 32 item scale that measures the degree to which an individual has had experiences that can be considered mystical during their lifetime. Participants rate their level of agreement with items such as “I have had an experience which I was unable to express adequately through language” on a 5-point Likert scale (from “definitely true” to “definitely not true”). We used the HMS total score in our analyses.

**Personality: M5P Personality Questionnaire (M5P) [74]**. The M5P is a 50 item, freely accessible measure with subscales for each domain of the 5-factor model of personality. Participants rate their level of agreement with items such as “I make friends easily” on a 5-point Likert scale (from “inaccurate” to “accurate”). We used the five personality subscale scores in our analyses: *Extraversion*, *Agreeableness*, *Conscientiousness*, *Neuroticism*, and *Openness*.

**Absorption: Tellegen Absorption Scale (TAS) [75]**. The TAS is a 34 item scale that measures disposition toward intense imaginative experiences, and a capacity to experience peak-like altered states of consciousness. Participants make true/false ratings for items such as “Sometimes I can change noise into music by the way I listen to it”. We used the TAS total score in our analyses.

**Creativity: Creative Personality Scale (CPS) [76]**. The CPS is comprised of two parts. General creativity is assessed through 20 items such as “I do unexpected things”, scored on a 5-point Likert scale (from “very inaccurate” to “very accurate”). Specific creativity is assessed through 10 items such as “How creative are you in the area of mathematics”, scored on 5-point Likert scale (from “not at all” to “extremely”). A total CPS score was calculated by summing the general and specific components. This total score was used in our analyses.

**Agency: Sense of Agency Rating Scale (SOARS) [77]**. The SOARS is a 10 item questionnaire with two subscales that measure distinct aspects of the sense of agency: *Involuntariness*, the sense that events that occur are externally generated; and *Effortlessness*, feelings of actions occurring easily and spontaneously. Participants rate their level of agreement with items such as “my experiences and actions were under my control” on a 7-point Likert scale (from “strongly disagree” to “very strongly agree”). We used the general form of the scale reported in Pritchard, Zopf, Polito, Kaplan and Williams [78]. *Involuntariness* and *Effortlessness* subscale scores were used in our analyses.

#### Debrief questions

Finally, at the conclusion of the study we asked participants a number of exploratory debrief questions, based on the psilocybin research protocol of Griffiths et al. [30]. Specifically, participants gave Likert ratings for the following questions: *Q1*. *How personally meaningful were your experiences microdosing during this study*? (from 1 = ‘No more than routine, every day experiences’ to 8 = ‘The single most meaningful experience of my life’); *Q2*. *Indicate the degree to which your experiences microdosing during this study were spiritually significant to you* (from 1 = ‘Not at all’ to 6 = ‘The single most spiritually significant experience of my life’); and *Q3*. *Do you believe that your experiences microdosing during this study have led to change in your current sense of personal well-being or life satisfaction*? (from -3 = ‘Decreased very much’ to +3 = ‘Increased very much’). At the conclusion of all measures participants also left brief unstructured comments about their experience in this study.

## Results

### Microdosing characteristics

Ninety-eight participants sent 1792 daily reports throughout the study, including 489 reports of microdosing days. Of these reports 3 were excluded due to doses much higher than all other reported microdoses (one participant reported a dose of 5 grams of psilocybin, and two participants reported doses of 150 micrograms of LSD); in addition, a further 8 records were excluded as participants reported concurrent use of other substances at higher doses in addition to microdosing on that day (substances included psilocybin, cocaine, DMT, LSD, amphetamines, MDMA and GHB). 478 reports of microdosing days were retained.

Participants reported microdosing with a wide range of substances. Reported substances included common psychedelics such as LSD (48.1% reports), psilocybin (47.1% daily reports), and mescaline (including synthetic mescaline and San Pedro cactus: 2.1% reports), but also a range of novel serotonergic psychedelics such as 4-hydroxy-N-methyl-N-ethyltryptamine (4-HO-MET; 1.0% reports), dimethoxybromoamphetamine (DOB; 0.8% reports), 4-chloro-2,5-dimethoxyphenethylamine (2C-C; 0.2% reports), 2,5-dimethoxy-4-methylphenethylamine (2C-D; 0.2% reports), 2,5-dimethoxy-4-ethylphenethylamine (2C-E; 0.2% reports) and morning glory seeds (i.e., lysergic acid amide, LSA; 0.2% reports). Reported doses for each substance are shown in Table 2. Doses are reported based on numerical recoding of participants textual reports. Some reports of LSD doses were in a format such as “1/10th dose”, in such cases we estimated typical doses as 100ug. For psilocybin, some participants reported “1 small shroom”, we estimated this to be .1g.

**Table 2:** Reported dosage by substance. Note that units of measurement vary across substances.

Substance	N	Mean Dose	Unit	SD	Min	Max
LSD	230	13.5	micrograms	8.5	1.4	50.0
Psilocybin	225	0.3	grams	0.3	0.1	1.5
Mescaline (Organic)	8	2.6	grams	2.7	0.1	6.0
4-HO-MET	5	4.0	milligrams	2.0	1.0	6.0
DOB	4	50.0	micrograms	25.8	20.0	80.0
Mescaline (Synthetic)	2	10.0	milligrams	0	10.0	10.0
2C-C	1	50.0	milligrams			
2C-D	1	5.0	milligrams			
2C-E	1	3.0	milligrams			
LSA	1	1.5	grams			

Participants reported a high level of certainty that their dosage estimates were accurate. On a scale from 1 (not confident at all) to 7 (completely confident), the mean confidence rating was 5.9 (SD = 1.4). Only 3 participants (5.1%) reported confidence in their dose estimates as less than 4.

Sixty-three participants completed baseline and post-study measures. 20 of these participants (31.7%) had never microdosed prior to taking part in the study, 12 (19.1%) 1–5 times prior, 10 (15.9%) 6–10 times prior, 3 (4.8%) 11–20 times prior, 9 (14.3%) 21–50 times prior, and 9 (14.3%) more than 50 times prior. 16 participants (25.8%) reported using psychedelics at higher doses, and 6 participants (9.7%) reported using MDMA at least once during the study period. Participants microdosed 5.0 times on average during the study (SD = 3.6, range 1–19 occasions). The mean time between doses was 6.7 days (SD = 4.8, range 1–34 days).

### Daily ratings

We analysed daily ratings from 98 participants using a linear mixed-effects model. Our aim in this analysis was to determine the effect of microdosing on the day that participants ingested a dose and the two days following dosing. We calculated the mean of each daily measure (*Connectedness*, *Contemplation*, *Creativity*, *Focus*, *Happiness*, *Productiveness*, and *Wellness*) on dosing days (Day0), as well as the day immediately following dosing (Day1), and the second day after dosing (Day2). A Baseline score was calculated as the mean rating for all other days within the study period (i.e., the mean of scores from all days apart from dosing days and the two days following dosing). We used R and nlme [79] to perform a mixed effects analysis of the effects of microdosing over time. Day type (Baseline, Day0, Day1, Day2) was entered as a categorical fixed effect. Intercepts for subjects were the only random effects. We were interested in changes in daily ratings on dosing days (Day0) and the days following dosing (Day1, Day2) compared to baseline ratings. Visual inspection of residual plots did not reveal any obvious deviations from homoscedasticity or normality. To ensure a balance of statistical power and also to minimise type II error, we treated each daily measure as a separate family and corrected for multiple comparisons within each measure using the Holm-Bonferroni adjustment [80].

**Fig 1:**
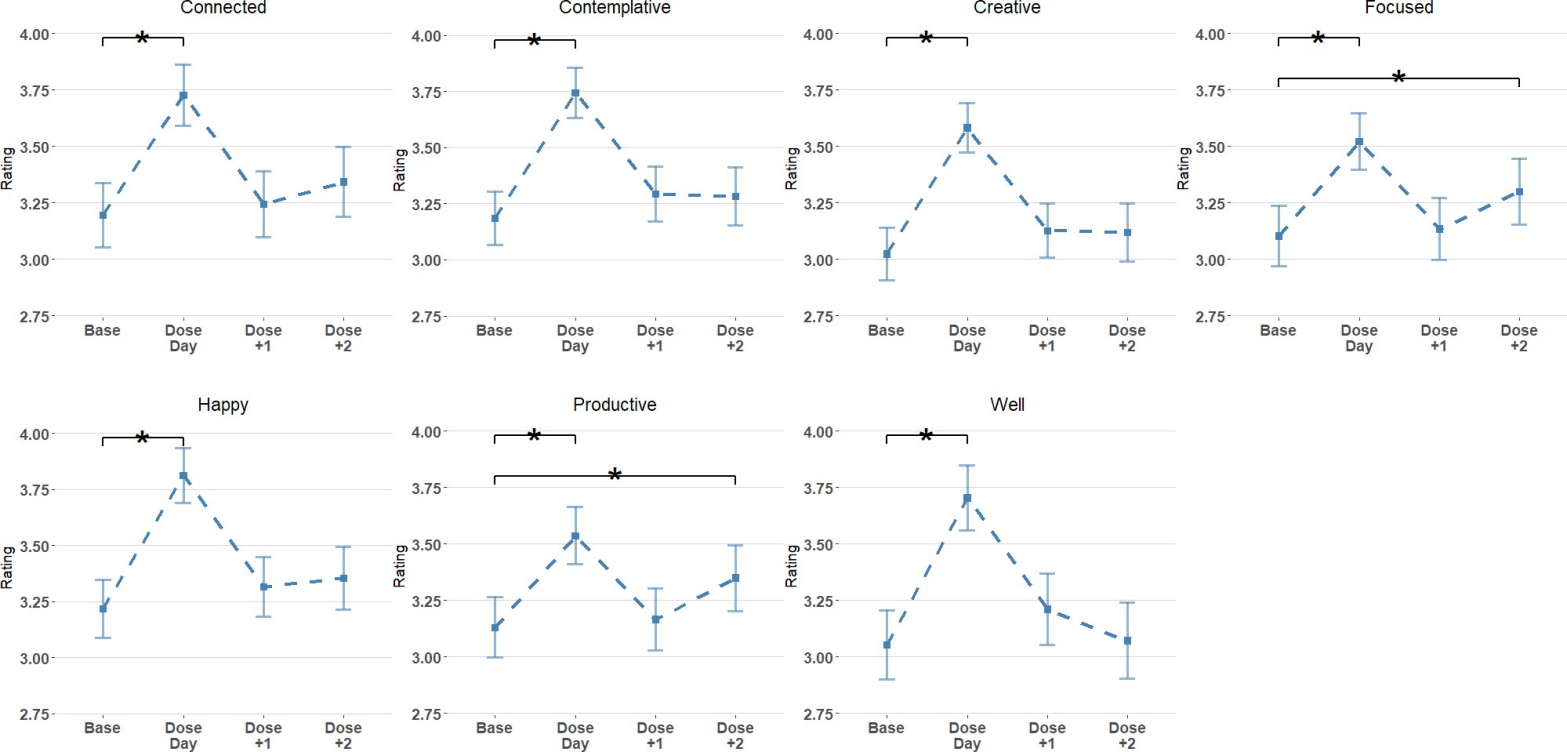
Daily ratings for each variable at Baseline, Dose Day (dosing day), Dose+1 (the day after dosing), and Dose+2 (2 days after dosing). Error bars show 95% confidence intervals. * indicates a significant difference from baseline (adjusted *p* < .05).

Fig 1 shows the pattern of results for all daily ratings. Table 3 reports the mean and standard deviation of each rating, as well as t-tests and significance for contrasts comparing each day category (Day0, Day1, Day2) to Baseline. There was a significant increase from Baseline on dosing days for all measures (all *p* < .001). This effect was not maintained on the day following dosing for any measures. Scores for *Focused* (*t* = 2.31, p = .044) and *Productive* (*t* = 2.61, *p =* .019) increased slightly two days following dosing and were significantly higher than baseline scores. As an indicator of effect size, we report the conditional coefficient of determination (*R*^*2*^_*C*_) for each rating, as implemented in the R package *MuMIn* [81,82]. We note that estimating effect sizes in mixed models is problematic as multiple R-squared measures have been proposed and there is little agreement among researchers as to the best procedure to follow [83]. The R^2^_C_ estimates provided here give a relative indication of the variance explained by each model, but should be interpreted with caution. Table B in S1 File shows the full model summary for daily ratings.

**Table 3:** Mean (SD) and contrast tests for daily ratings.

	Baseline	Dosing Day [Day0]	Day0 v Baseline	Dosing Day +1 [Day1]	Day1 v Baseline	Dosing Day +2 [Day2]	Day2 v Baseline	*R*^*2*^_*C*_
**Connected**	3.2 (0.5)	3.7 (0.7)	*t* = 6.66, *p* < .001*	3.2 (0.7)	*t* = 0.60, *p* = .552	3.3 (0.7)	*t* = 1.68, *p* = .188	0.427
**Contemplative**	3.2 (0.5)	3.8 (0.6)	*t* = 8.33, *p* < .001*	3.3 (0.6)	*t* = 1.53, *p* = .254	3.3 (0.6)	*t* = 1.32, *p* = .254	0.447
**Creative**	3.0 (0.4)	3.6 (0.6)	*t* = 8.11, *p* < .001*	3.1 (0.6)	*t* = 1.42, *p* = .312	3.1 (0.5)	*t* = 1.26, *p* = .312	0.397
**Focused**	3.1 (0.5)	3.5 (0.7)	*t* = 5.36, *p* < .001*	3.1 (0.6)	*t* = 0.38, *p* = .703	3.3 (0.7)	*t* = 2.31, *p =* .044*	0.355
**Happy**	3.2 (0.5)	3.8 (0.6)	*t* = 7.89, *p* < .001*	3.3 (0.6)	*t* = 1.23, *p* = .220	3.3 (0.7)	*t* = 1.64, *p* = .204	0.406
**Productive**	3.1 (0.5)	3.5 (0.6)	*t* = 5.32, *p* < .001	3.2 (0.7)	*t* = 0.44, *p* = .664	3.3 (0.8)	*t* = 2.61, *p =* .019*	0.387
**Well**	3.0 (0.5)	3.7 (0.8)	*t* = 7.12, *p* < .001*	3.2 (0.7)	*t* = 1.64, *p* = .205	3.1 (0.8)	*t* = 0.19, *p* = .850	0.372

p-values are adjusted using the Holm-Bonferroni adjustment for three comparisons within each family.

* p < 0.05

### Long term measures

Our second set of analyses investigated changes across nine domains of psychological functioning (mood, attention, wellbeing, mindfulness, mystical experience, personality, absorption, creativity, agency) from baseline (when first signing up for the study) to post study (after 6 weeks of microdosing) for 63 participants. We calculated omega total scores as a measure of internal consistency for all scales [84]. Internal consistencies were good at both baseline and post study for all measures (all ω_t_ > .75, see Table C in S1 File). Participants in this study differed in their prior experience microdosing, and also in their frequency of microdosing during the study period. We generated linear effects models to map these influences on each measure over the study period. Our model included *Time* (baseline vs post study) as a categorical fixed effect. We also coded prior microdosing experience (*Experience)* as a categorical fixed effect with two levels: no prior experience (n = 19) and some prior experience (n = 43). Number of doses during the study (*Doses*) was entered as a continuous fixed effect. Intercepts for subjects were the only random effects. Our model also included an interaction of *Time x Experience* and an interaction of *Time x Doses*. There were three critical analyses in our model: the main effect of *Time*, and the two interactions (*Time x Experience* and *Time x Doses*). We were not interested in the main effects of either *Experience* or *Doses* (i.e., we primarily wanted to see how *Experience* and *Dose* influenced changes in scores from baseline to post-study, rather than the overall influence of these main effects averaged across timepoints. Nevertheless, a full model summary including these main effects is shown as Table D in S1 File).

**Fig 2:**
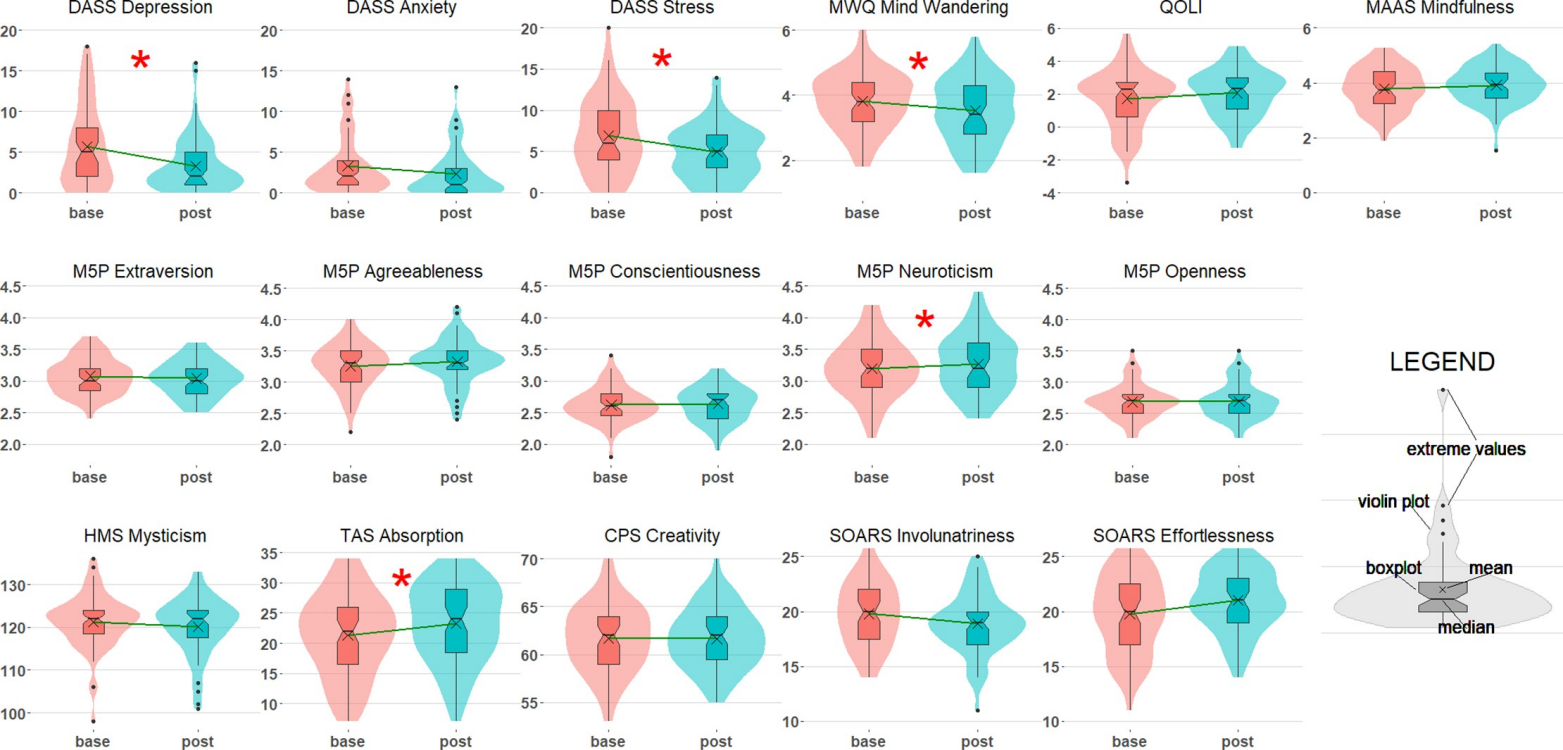
Baseline and post study scores. These panels show distributions and summary statistics for each long term measure at baseline and post study. Boxplots show median and inter quartile ranges for each variable. Violin plots show the distribution of responses. The green line plots the difference between means from baseline to post study. * indicates a significant difference from baseline to post study (adjusted *p* < .05).

Visual inspection of residual plots did not reveal any obvious deviations from homoscedasticity or normality. Each subscale in these analyses was treated as a separate family and our three critical analyses were corrected using the Holm-Bonferroni adjustment. Fig 2 shows the pattern of changes for all long term measures. Table 4 reports the mean and standard deviation for each measure at baseline and at conclusion of the study. We also report t tests, corrected p-values for our three critical tests (main effect of *Time*, *Time x Experience* interaction, *Time x Doses* interaction), and *R*^*2*^_*C*_ as an estimate of the variance explained by each model.

**Table 4:** Long term battery scores. This table shows baseline and post study means and standard deviations for each measure. Also shown are test statistics for the three critical analyses: the main effect of *Time*, and interactions of *Time x Experience* and *Time x Dose*. p-values are adjusted using Holm-Bonferroni correction for three comparisons. *R*^*2*^_*C*_ estimates the variance explained by each model. A complete model summary table is included as Table D in S1 File.

				Time		Time x Exp		Time x Doses		*R*^*2*^_*C*_
		Base (SD)	Post (SD)	*t*	*p*	*t*	*p*	*t*	*p*	
**DASS**										
	Depression	5.7 (5.0)	3.3 (3.4)	-3.97	**0.001**	-0.53	1.000	0.44	1.000	0.469
	Anxiety	3.3 (3.5)	2.3 (2.8)	-1.95	0.169	0.36	1.000	0.20	1.000	0.440
	Stress	6.9 (4.5)	4.9 (3.2)	-3.36	**0.004**	0.11	1.000	0.67	1.000	0.438
**Mind Wandering**										
	MWQ	3.8 (0.9)	3.5 (1.0)	-2.49	**0.047**	-0.06	1.000	0.19	1.000	0.595
**Quality of Life**										
	QOLI Total	1.7 (1.8)	2.1 (1.4)	1.41	0.496	-0.73	1.000	-0.28	1.000	0.650
**Mindfulness**										
	MAAS	3.8 (0.8)	3.9 (0.7)	1.32	0.573	0.13	1.000	2.00	0.152	0.510
**Mystical Experiences**										
	HMS Total	121.3 (6.4)	120.2 (6.7)	-1.08	0.850	0.46	1.000	0.01	1.000	0.515
**Personality**										
	Extraversion	3.1 (0.3)	3 (0.3)	-1.03	0.926	-0.09	1.000	-1.22	0.687	0.591
	Agreeableness	3.3 (0.4)	3.3 (0.4)	1.97	0.161	1.02	0.929	1.26	0.634	0.642
	Conscientiousness	2.6 (0.3)	2.6 (0.3)	1.25	0.645	2.38	0.062	1.41	0.488	0.510
	Neuroticism	3.2 (0.5)	3.3 (0.5)	2.70	**0.027**	1.86	0.203	0.46	1.000	0.844
	Openness	2.7 (0.3)	2.7 (0.3)	0.31	1.000	0.49	1.000	1.16	0.746	0.557
**Absorption**										
	TAS Total	21.3 (6.8)	23.3 (6.5)	4.46	**< 0.001**	1.26	0.643	-0.84	1.000	0.855
**Creativity**										
	CPS Total	61.7 (3.6)	61.7 (3.2)	-0.55	1.000	-1.4	0.498	0.70	1.000	0.427
**Sense of Agency**										
	Involuntariness	19.8 (3.0)	18.9 (2.7)	-2.18	0.099	-0.52	1.000	-0.56	1.000	0.354
	Effortlessness	19.8 (3.5)	21 (2.9)	2.31	0.072	-0.32	1.000	-0.91	1.000	0.321

We found a main effect of *Time* (i.e., change from baseline to post-study scores) for a number of measures. Participants reported significant improvements in mood: specifically, *Depression* (*t* = -3.97, *p =* .001) and *Stress* (*t* = -3.36, *p* = .004) decreased during the study period, indicating participants experienced improvements in their mental health after microdosing. *Mind Wandering* decreased significantly during the study period (*t* = -2.49, *p* = .047) indicating that participants were better able maintain focus after microdosing. *Absorption* increased significantly over the study period (*t* = 4.46, *p* < .001), indicating that participants became more involved in imaginative experiences after microdosing. The only personality change indicated by these data was a slight increase in *Neuroticism* (*t* = 2.70, *p* = .027), indicating that participants tended to experience more negative emotions after microdosing. There were no significant interactions of *Time* x *Experience* or *Time* x *Doses*, suggesting that changes in psychological functioning were not influenced by prior microdosing experience or by the frequency of doses taken during the study period.

There was a trend toward an increase in *Effortlessness* scores, and to a lesser a degree, a trend toward an increase in *Involuntariness*. This indicates that microdosing may have influenced participants’ sense of agency such that actions felt more internally generated (reduction in *Involuntariness*) and also more spontaneous (increase in *Effortlessness*). There was also a trend toward an interaction of *Time* x *Doses* for *Conscientiousness*, such that those participants who more regularly took microdoses during the study period scored higher. This finding may reflect that individuals with conscientious temperaments are those most likely to commit to a regular schedule when experimenting with microdosing. Each of these trends may indicate worthwhile directions for future research.

### Exploratory debrief questions

For ease of comparison, answers to Debrief Questions were rescaled on scale from 1–100. The majority of participants did not categorise their experience of microdosing during the study as particularly meaningful. The mean rating for *Q1*. *How personally meaningful were your experiences microdosing during this study*? was 38.95 (SD = 25.71). There were however a minority of participants who reported that microdosing during the study was extremely meaningful. Five participants (8.06%) reported that this period of microdosing was ‘among the 5 most meaningful experiences of my life’, and one participant (1.61%) claimed this period of microdosing was ‘the single most meaningful experience of my life’.

Similarly, the vast majority of participants did not report that microdosing during this study was spiritually significant. The mean rating for *Q2*. *Indicate the degree to which your experiences microdosing during this study were spiritually significant to you* was 31.69 (SD = 27.86). Again, there were a small number of participants who reported that the experience had considerable spiritual significance. Five participants (8.06%) reported that this period of microdosing was ‘among the 5 most spiritually significant experiences of my life’, and two participants (3.23%) claimed this period of microdosing was ‘the single most spiritually significant experience of my life’.

Finally, participants overall reported that microdosing increased feelings of personal well-being. The mean rating for *Q3*. *Do you believe that your experiences microdosing during this study have led to change in your current sense of personal well-being or life satisfaction*? was 78.55 (SD = 18.05). 54 participants (87.09%) reported that microdosing in this study slightly, moderately, or very much increased subjective well-being. However, this single item rating was not correlated with the post study QOLI Total score (which also measured wellbeing), *t*(57) = 1.62, *p* = 0.110.

## Study One discussion

Daily ratings provided a snapshot of participants’ subjective experiences on each day of the study. These ratings revealed that microdosing led to an increase across all psychological functions measured on dosing days, compared to baseline scores. For the most part, these increases were not maintained in the days following dosing, although subjective reports of *Focus* and *Productivity* were slightly increased two days after microdosing. These findings indicate that microdosing led to general increases in psychological functioning rather than specific effects. The pattern of results here is somewhat inconsistent with narrative accounts that claim that the effects of microdosing linger for multiple days [8]. However, in the context of media stories which promote microdosing as a tool for ambitious professionals [16,18], it is notable that two scores that could be related to increased capacities at work (i.e., *Focus* and *Productivity)* did show an effect two days following dosing.

Detailed long term questionnaires revealed several variables that changed during the microdosing period. Broadly these effects could be characterised as improvements in mental health, and alterations in processes related to attention.

This study focused on non-clinical effects of microdosing and participants with any self reported mental illness were asked not to take part. As such, ratings on the DASS scale were relatively low at baseline. Nevertheless, depression and stress ratings both decreased significantly over the course of the study, consistent with reports that microdosing benefits general mental wellbeing [7].

Participants also reported significant reductions in mind wandering. Reduced mind wandering may lead to a number of benefits that meet the aims of microdosers, including reduced distractibility and increased capacity to focus on the task at hand [85]. Reduced mind wandering has also been linked with improved educational outcomes [86], and greater levels of happiness [87]. At the neural level, reduced mind wandering has been associated with a reduction in activity in the default mode network [88]–a phenomenon that has also been reported following ingestion of psychedelics at higher doses [50].

Participants additionally reported increases in absorption. Absorption has been described as a type of focused attention characterised by deep involvement with particular aspects of subjective experience [89]. Individuals with high levels of trait absorption are likely to report intense engagement with nature and aesthetic involvement with art [90]. These characteristics are consistent with descriptive reports from participants in the current study (see Table 5).

**Table 5:** Participant comments demonstrating experiences of absorption, intense emotions and unease.

Comments demonstrating experiences of absorption	
	*“[COMMENT D346] I had a very reflective day*. *I felt blissfully connected with nature and came up with optimistic ideas for the future*.*”*
	*“[COMMENT D178] I was finding that all music inspired unique mental imagery*. *Much like each frequency was creating a unique figure in my mind’s eye*, *very fascinating*.*”*
	*“[COMMENT D175] I had an interesting experience sitting on my porch at my house in the forest*. *All the sounds in the forest became an orchestra*, *it was beautiful*, *layered and rhythmic*.*”*
	*“[COMMENT D439] I felt a lot more connected to nature*, *there are dogs where I am working*. *I'm not totally comfortable around dogs*, *but I sat with 3 of them a while*, *we were still together while they lay in the sun*, *I felt connected and deeply grateful for their trust and support*.*”*
	*“[COMMENT D183] We went to see a band play*, *and I found I was able to connect to the music in a deeper way”*
Comments demonstrating experiences of intense emotions	
	*“[COMMENT L2] Microdosing has a significant impact on my ability to get in touch with what is going on deep inside*. *Although this is not always a pleasant experience*, *I have a strong feeling that psilocybin helps to reveal what I need to see in myself and the world*.*”*
	*“[COMMENT L3] I was surprised to find myself crying a lot throughout the study despite the fact that I wasn't going through anything typically difficult”*.
Comments demonstrating unease about microdosing	
	*[Comment D210] On a microdose I sometimes feel weird or alien to myself and others*. *And another negative is that all emotions get amplified*. *So whenever I feel down or not loved the microdose makes it even harder*.
	*[Comment D308] The first hour was productive but then doubt*, *confusion*, *and uncertainty crept in*. *Found it difficult to make decisions*, *felt very unassertive*.

Notably, absorption is closely linked to an individual’s capacity to experience altered states of consciousness and has been identified as the most important trait predictor of boundary dissolution and visual alterations in studies using higher doses of psilocybin [91]. Furthermore, Ott, Reutger, Hennig and Vait [92] showed that individuals with a particular genetic polymorphism linked to stronger binding potential for the 5-HT2A receptor (a key target of serotonergic psychedelics) scored higher on measures of absorption. These previous studies indicate that individuals with higher pre-existing levels of trait absorption are more likely to experience stronger effects from psychedelic substances. The novel implication from our findings is that regular microdosing may also increase individual’s capacity for absorption.

The idea of systematically increasing absorption is not new. Luhrmann, Nusbaum and Thisted [93] have argued that the rituals and practices of some religious groups are designed to enhance trait levels of absorption and that this increased capacity leads practitioners to have more intense religious experiences. It may be that microdosing has a comparable effect, whereby regular experience of the subtle alterations in consciousness associated with microdosing slowly increase the sensitivity and responsiveness of microdosers to future consciousness alterations.

Participants in this study also reported a small increase in trait neuroticism. Primary personality traits are typically considered very stable constructs, so any alteration over such a short period is surprising. An increase in neuroticism is somewhat inconsistent with the results showing reductions in standardised measures of mental health reported above. This increase in neuroticism may reflect an overall increase in the intensity of emotions (both positive and negative) experienced during periods of microdosing. Reports of intense emotions were common in participants’ comments, see Table 5 for examples. It may be that as participants become less distracted (i.e., experience reduced mind wandering) and more absorbed in their immediate experience, they are more able to identify and process negative emotions.

Many of the domains of psychological functioning investigated did not change during six weeks of microdosing. Specifically, we did not identify any changes on measures of mindfulness, mystical experience, positive personality traits, creativity, sense of agency or overall quality of life. These results do not support recent findings showing an increase in creative thinking following microdosing [21]. However, constructs such as creativity [94] and mindfulness [95], are particularly difficult to assess using rating scales. As such, these null findings may reflect insufficient sensitivity of these measures.

## Study Two

Some of the domains which did not change in Study One (e.g., creativity and quality of life) are very commonly described in media stories on the effects of microdosing [96,97]. Conversely, some domains that did change in Study One (e.g., absorption, neuroticism) are not typically mentioned in media stories on microdosing. This indicates that common media narratives may not match up with people’s experiences when measured in a systematic way. In Study Two we were interested in how the prominence of microdosing in popular media has led to specific expectations about the effects of microdosing, and how these expectations matched or differed from our findings in Study One.

Expectations can affect human behaviour in a range of ways, including influencing hypnotic responses [98] increasing sensory attenuation [99], and enhancing mindfulness [100]. Expectations can also have particularly profound impacts on drug effects, even amongst individuals with no prior experience of a particular substance [101]. Study One recruited participants from online communities that share and discuss media and personal accounts of microdosing that are mainly very positive. Participation in these communities may lead to specific expectations that shape individuals’ experiences of microdosing. Most of the early research on microdosing has not included a control group or explicitly investigated participant expectations (although see [23]). It was not practicable for us to directly test placebo responses in this research, but in Study Two we investigated expectations held by members of online microdosing communities to see if these may help explain the experiences of microdosers in Study One.

We recruited a large sample from the same population as Study One, and asked participants to rate their expected changes across each of our domains of interest. Participants were asked not to take part if they had participated in Study One. This study consisted of a single online questionnaire about microdosing expectations.

## Method

### Participants

Participants were recruited through the same online networks, and with the same inclusion and exclusion criteria as Study One. Study Two commenced on 3^rd^ April 2017. We did not post any new recruitment notices for this study. We simply updated our study webpage so that from this commencement date, any individuals clicking on links we had previously posted online were shown information for Study Two. The study was open from April 2017 to July 2017. 501 individuals started the study. 238 records were incomplete and therefore excluded, leaving 263 participants who completed the microdosing expectations questionnaire. Of these, 152 participants (57.8%) reported never having microdosed previously and 111 (42.8%) had had experience microdosing. 162 participants (61.6%) were male, 99 (37.6) were female, and 2 (0.8%) did not specify gender. Again, participants nominated age ranges rather than reporting exact ages. 82 (31.2%) participants were aged 18–25, 78 (29.7%) were aged 26–35, 50 (19.0%) were aged 36–45, 33 (12.5%) were aged 46–55, and 20 (7.6%) were aged 56+. Participants were mainly from the USA (64.4%), Australia (15.3%), and Canada (6.5%). The sample was highly educated with 62.3% participants having completed tertiary education. 22.1% participants were students and 66.9% were working full time or part time.

Although participants for both Study One and Study Two were drawn from the same population, there were some demographic differences between these samples. Specifically, Fisher’s exact test revealed that the samples differed in age (*p* = 0.035). This appears to driven by a greater proportion of participants in the 26–65 age bracket in Study One, whereas participants were more evenly spread through across age brackets in Study Two. The samples also differed on country of residence (*p* < .001), with a greater proportion of participants residing in the United States in Study Two (64.4%) compared to Study One (17.7%). The samples did not differ on gender (*p* = 0.064), education (*p* = 0.586), or employment status (*p* = 0.550).

### Procedure

As a way of indexing participants’ expectations about the effects of microdosing we generated a new scale, consisting of items that related to each of the subscales analysed in Study One. We constructed a statement that described the type of experience tapped by each subscale. For example, for the DASS Depression subscale, our item was “feelings of depression”. Items for all subscales are shown in Table 6. Participants were given the following instructions:

*What happens when people microdose? Imagine that you personally were to regularly microdose for a period of 6 weeks. If you were to do that, do you think the frequency of each of the following experiences would be likely to decrease*, *stay the same, or increase over that time period?*

**Table 6:** Expectation items that were rated for each subscale; weighted expectation scores (positive scores indicate an expectation that this subscale would increase, negative scores indicate an expectation that this subscale would decrease); one-sample t-test results comparing weighted expectations score to 0; rank order of expectation effects (based on weighted expectation scores); rank order of effects found in Study One (based on t value for main effect of *Time* in Study One).

Variable	Expectation Scale Item (Target Experience)	Weighted Expectation (weighted variance)	*t*(262) [difference from 0]	*p*	*d*	Expectation Rank	Study 1 Rank
**CPS: Creativity**	Feeling that you have a creative personality	+.94 (.07)	59.44	< .001	3.67	1	9
**MAAS: Mindfulness**	Becoming absorbed and involved in imaginative and sensory experiences	+.92 (.09)	49.07	< .001	3.03	2	6
**QOLI: Quality of Life**	Overall satisfaction with life	+.92 (.09)	48.71	< .001	3.00	3	5
**M5P: Openness**	Feeling curious about new experiences	+.87 (.12)	40.91	< .001	2.52	4	8
**HMS: Mystical Experience**	Experiences of connection and unity with all things	+.86 (.12)	40.62	< .001	2.50	5	11
**M5P: Agreeableness**	Feeling agreeable and cooperative	+.76 (.21)	26.89	< .001	1.66	6	4
**M5P: Extraversion**	Feeling extroverted (i.e., feeling energetic, assertive and social)	+.75 (.25)	24.58	< .001	1.52	7	10
**M5P: Conscientiousness**	Feeling disciplined and achievement focused	+.73 (.27)	22.58	< .001	1.39	8	7
**TAS: Absorption**	Becoming absorbed and involved in imaginative and sensory experiences	+.66 (.30)	19.52	< .001	1.20	9	1
**SOARS: Effortlessness**	Feeling that actions occur without effort	+.46 (.36)	12.48	< .001	0.77	10	3
**SOARS: Involuntariness**	Feeling that actions occur involuntarily	-.10 (.39)	-2.60	.010	0.16	11	13
**MWQ: Mind Wandering**	Tendency for your mind to wander (i.e., the tendency for your focus on a task to be interrupted by unrelated thoughts).	-.22 (.65)	-.4.48	< .001	0.28	12	14
**M5P: Neuroticism**	Feeling emotionally unstable (i.e., feeling angry, anxious or vulnerable)	-.72 (.33)	-20.18	< .001	1.24	13	2
**DASS: Anxiety**	Feelings of anxiety	-.76 (.29)	-22.89	< .001	1.41	14	12
**DASS: Stress**	Feelings of stress	-.84 (.18)	-31.76	< .001	1.96	15	15
**DASS: Depression**	Feelings of depression	-.88 (.18)	-33.93	< .001	2.09	16	16

Participants’ frequency expectations were scored on a 3-point scale (decrease = -1, stay the same = 0, increase = +1). In addition, participants were asked to rate their confidence in each of these directional predictions (from 1 = not at all sure, to 5 = extremely sure).

The study took approximately 15 minutes to complete and there were no incentives for taking part.

## Results

We calculated a weighted expectation score for each item using the R package *Hmisc* [102]. Weights were calibrated such that the maximum confidence rating carried twice the weight of the minimum confidence rating (i.e., a confidence rating of 1 was weighted as 1.00, 2 was weighted as 1.25, 3 was weighted as 1.5, 4, was weighted as 1.75, and 5 was weighted as 2.00). The derived weighted expectation scores ranged from -1 to +1. The rank orders of weighted expectation scores for naïve and experienced microdosers were highly correlated (*Kendall’s τb* = .934, *p* < .001), so we report results based on the combined dataset (Table E in S1 File shows weighted expectation scores for naïve and experienced participants separately). This strong similarity between naïve and experienced participants indicates that individuals in this population held consistent expectations about the effects of microdosing regardless of their personal experience.

We performed a one-sample weighted t-test to determine whether expectation scores differed from 0 for each subscale (i.e., whether participants had a significant expectation that the frequency of target experiences would increase or decrease). Participants’ (*n* = 263) expected that all variables would change following microdosing (all p < = .01; see Table 6). Specifically, depression, anxiety, stress, mind wandering, neuroticism, and involuntariness were expected to decrease. Quality of life, mindfulness, mysticism, extraversion, agreeableness, conscientiousness, openness, absorption, creativity, and effortlessness were expected to increase. Expectations in Study Two matched the direction of each of the significant findings in Study One, with the exception of neuroticism.

We ranked participants’ expectations for each variable, based on the mean weighted expectation scores. These ranks take into account the expected direction of change such that the first rank indicates the variable most expected to increase, and the last rank indicates the variable most expected to decrease. Table 6 also shows the rank order of effects found in Study One (based on t values for the main effect of *Time*, and also ordered from the variable that increased the most through to the variable that decreased the most). A Kendall's tau-b correlation of these two rank orderings did not find evidence of an association between expectations in Study Two and effects in Study One, *τb* = .283, *p* = .139. In other words, participants’ ranked expectations about which variables would change were unrelated to the actual rankings of variables that did change in Study One. Many of the variables most expected to change in Study Two actually showed relatively small changes in Study One. Conversely most of the variables that showed the largest changes in Study One were not those that participants expected would change. This outcome is not consistent with an expectancy bias explanation for the Study One findings.

## Study Two discussion

Participants in Study Two had strong expectations about the various effects of regular microdosing. Participants expected that all of the experiences they were asked to rate would either increase or decrease in frequency. Most changes found in Study One followed the expected direction of change in Study Two (i.e., decreased depression, decreased stress, decreased mind wandering, and increased absorption). The only direct contradiction between expected and actual effects of microdosing was that participants in Study Two expected that neuroticism would decrease following microdosing, whereas a small but significant increase was found in Study One.

Participants in Study Two did have many predictions that, although not directly contradicted were not supported by the evidence from Study One. Participants in Study Two expected all measures would change following microdosing but that is not what we found. In particular, participants in Study Two had very strong predictions that creativity, wellbeing, and mindfulness would increase (weighted expectation scores all > .90), but none of these measures showed significant increases in Study One. Overall there was no evidence of an association between participants’ expectations about which variables would change due to microdosing in Study Two and the effects of microdosing found in Study One.

## General discussion

This was exploratory research that investigated people’s experience of and attitudes toward microdosing. Study One showed that, in the short term, microdosing led to an immediate boost across a range of psychological variables but that these effects were (mostly) not sustained over multiple days. Longer term, we found evidence that microdosing led to improved mental health, altered attentional capacities (reduced mind wandering and increased absorption), and increased neuroticism.

Study Two showed that amongst people who are interested in microdosing there are strongly held beliefs that microdosing can impact a wide range of psychological variables. A substantial majority of media reports on microdosing present the practice in glowing and positive terms. This may have led to a perception of microdosing as a general panacea that is able to improve virtually all aspects of an individual’s life. It is not clear the degree to which these expectations influence individuals’ interpretation and reports of their microdosing experiences.

Taken together these findings paint an intriguing picture. We found clear changes in a small set of psychological variables: decreased depression and stress; decreased mind wandering; increased absorption; and increased neuroticism. Notably, these variables were not those that participants most expected to change. If the current findings were entirely due to expectation, then we should have seen changes in those variables that are most commonly discussed in media and online accounts of microdosing, and in those variables rated highest in Study Two. In fact several of the most commonly discussed effects of microdosing and the effects most expected to change (creativity, wellbeing, mindfulness) showed no evidence of alteration whatsoever. This suggests that the longer term changes we identified were unlikely to be due to expectation.

On the other hand, although we did identify clear short term changes following each microdose in the daily analyses, the longer term changes identified in Study One were unrelated to the total number of doses participants ingested during the study period and also unrelated to participants’ prior microdosing experiences. This surprising lack of a relationship between the overall quantity of microdoses and the degree of subjective effects is a reason to interpret these findings cautiously. At face value this suggests that any engagement with microdosing, whether a single dose or relatively frequent dosing, can impact the variables we identified. This may be the case, but it is also possible that participants’ self reports of dosage and frequency in this study were not precise enough to accurately characterise dose related effects.

Overall, these findings suggest several disconnects between the popular narrative around microdosing and the experience of microdosers in this sample. Participants in Study One microdosed less often than is recommended in most online protocols and did not report that many of the immediate effects of microdosing lasted beyond the day of dosing. Although popular accounts of microdosing describe sustained boosts in productivity and creativity [16–18], the longer term effects we identified mainly involved reduced mental distress and changes in constructs such as absorption and mind wandering that are not as commonly discussed. This suggests that microdosing may lead to more subtle changes characterised by improvements in mental stability, the capacity to sustain attention and increased ability to become engaged in intense imaginative experiences.

The most surprising finding was that neuroticism also appeared to increase following microdosing. This is not something that is discussed in popular accounts of microdosing and was not what participants expected in Study Two. This highlights an important and under discussed aspect of microdosing: not everyone has a positive experience. Although the majority of participants’ comments were positive (and even glowing), there were a subset of comments that reflected unease about microdosing (see Table 5). In a context of considerable hype around the practice of microdosing, particularly with regards to it’s potential as a business tool, it important to acknowledge that microdosing may not be universally beneficial. These findings highlight the need for further research into the full range of microdosing effects (positive and negative) and also for investigations into subtypes of individuals who may particularly benefit from or be adverse to the practice.

### Strengths, limitations and future directions

This was very much a preliminary and exploratory study of microdosing, and there are clear limitations to the study design. This was a self reported observational study and as such, we relied on participants’ accuracy and honesty in their reports of doses and effects, and also on their continued responsiveness throughout the study period. Recruitment for this study occurred through online forums that were mainly very positive about the effects of microdosing. As such, these results may be affected by sampling bias, and may under represent individuals with negative or ambivalent experiences of microdosing. Furthermore, as might be expected in an observational study of individuals who are interested in psychedelics, there was some concurrent use of higher dose psychoactive substances and non psychedelic substances that may have had some influence on these results. A better design would certainly be to conduct an experimental study with controlled doses of known substances and a placebo comparison condition. The legal and bureaucratic limitations around psychedelic research make the approval process for such a study prohibitive. As an alternative, in implementing the current design we aimed to demonstrate that it is possible to investigate the effects of psychedelic substances in a systematic observational paradigm, using an automated and anonymous communication system.

This study involved investigation of a wide range of psychological variables. We limited the likelihood of obtaining false positive results in two ways. First, in our long term analyses we minimised the number of subscales investigated by using only a total or mean summary score wherever practical. For example, the Tellegen Absorption Scale has six subscale scores but we analysed only the total absorption score. Second, in all of our analyses we used conservative adjustments to account for multiple comparisons within each domain. We acknowledge that we cannot draw strong conclusions from these exploratory findings but suggest that the effects identified are worthy of further investigation in future confirmatory research with specific hypotheses. In particular, it will be important to untangle the role of expectation, either by implementing a placebo condition or by investigating the direct influence of expectations on individuals’ subsequent microdosing experiences. Future research also needs to focus on better understanding the impacts of frequency and dosage.

It is clear from this research that there is a high level of popular interest in microdosing, that many people are engaged in this practice, and that there are strong expectations about the various effects that microdosing can have. The current findings suggest that popular accounts of the effects of microdosing may not match the experience of long term microdosers, and that promising avenues for future investigation are the impacts of microdosing on improved mental health, attentional capabilities, and neuroticism.

## Supporting information

S1 File Supporting information. (DOCX)
