# Supplementary material for: A systematic study of microdosing psychedelics
Source: PLoS One. 2019 Feb 6;14(2):e0211023. doi: 10.1371/journal.pone.0211023 (PMC6364961; doi:10.1371/journal.pone.0211023)
Supplement: S1 File — (DOCX) [file pone.0211023.s001.docx]

**Supplementary Materials**

*Data and additional materials are available at* [osf.io/6xfm8/](http://osf.io/6xfm8/)

Table A: Summary statistics for the Altered States of Consciousness Scale (Studerus, Gamma, & Vollenweider, 2010) and Persisting Effects Questionnaire (Griffiths, Richards, McCann, & Jesse, 2006) administered as part of the post-study battery in Study One.
Each subscale score is the mean of all items in that subscale. Items in the Altered State of Consciousness Questionnaire were scored from 1 to 100. Items in the Persisting Effects Questionnaire were scored from 1 to 8.

|  |  | **Mean** | **SD** | **Min** | **Max** |
| --- | --- | --- | --- | --- | --- |
| **Altered States of Consciousness Scale** | |  |  |  |  |
|  | Total ASC Score | 28.42 | 17.03 | 0.76 | 63.47 |
|  | Oceanic Boundlessness | 37.94 | 23.19 | 1.22 | 86.89 |
|  | Dread of Ego Dissolution | 12.52 | 12.18 | 0.00 | 47.81 |
|  | Visionary Restructuring | 32.71 | 21.69 | 0.56 | 83.28 |
|  |  |  |  |  |  |
|  | Experience of Unity | 36.97 | 27.58 | 0.00 | 90.80 |
|  | Spiritual Experience | 36.54 | 26.33 | 0.00 | 100.00 |
|  | Bliss | 46.56 | 28.97 | 0.00 | 100.00 |
|  | Insightfulness | 49.82 | 30.75 | 0.00 | 100.00 |
|  | Disembodiment | 21.70 | 26.47 | 0.00 | 100.00 |
|  | Impaired Control | 12.74 | 12.62 | 0.00 | 51.29 |
|  | Anxiety | 12.84 | 14.69 | 0.00 | 54.17 |
|  | Complex Imagery | 36.65 | 28.28 | 0.00 | 95.33 |
|  | Elementary Imagery | 29.12 | 28.36 | 0.00 | 96.33 |
|  | Synathesia | 21.98 | 26.06 | 0.00 | 86.67 |
|  | Changed Meaning | 40.50 | 26.74 | 0.00 | 96.67 |
| **Persisting Effects Questionnaire** | |  |  |  |  |
|  | Positive Attitudes | 2.56 | 0.42 | 1.00 | 3.59 |
|  | Negative Attitudes | 2.48 | 0.52 | 1.00 | 3.59 |
|  | Positive Mood | 2.94 | 0.56 | 1.25 | 4.00 |
|  | Negative Mood | 3.21 | 0.92 | 1.00 | 5.25 |
|  | Altruism | 2.13 | 0.55 | 1.00 | 3.38 |
|  | Antisocial | 2.13 | 0.57 | 1.13 | 3.38 |
|  | Positive Behaviour | 3.65 | 1.28 | 1.00 | 6.00 |
|  | Negative Behaviour | 3.42 | 1.26 | 1.00 | 6.00 |

Table B: Model Summary for Daily Ratings in Study One. p-values are corrected using the Holm-Bonferroni adjustment for three comparisons.

|  | **Intercept** | **Day0** | | | **Day1** | | | **Day2** | | |
| --- | --- | --- | --- | --- | --- | --- | --- | --- | --- | --- |
|  | ***b*** | ***b*** | ***t*** | ***p*** | ***b*** | ***t*** | ***p*** | ***b*** | ***t*** | ***p*** |
| Connected | 3.20 | 0.53 | 6.66 | **< 0.001** | 0.05 | 0.60 | 0.552 | 0.15 | 1.68 | 0.188 |
| Contemplative | 3.19 | 0.56 | 8.33 | **< 0.001** | 0.11 | 1.53 | 0.254 | 0.10 | 1.32 | 0.254 |
| Creative | 3.02 | 0.56 | 8.11 | **< 0.001** | 0.10 | 1.42 | 0.312 | 0.10 | 1.26 | 0.312 |
| Focused | 3.10 | 0.42 | 5.36 | **< 0.001** | 0.03 | 0.38 | 0.703 | 0.20 | 2.31 | **0.044** |
| Happy | 3.22 | 0.59 | 7.89 | **< 0.001** | 0.10 | 1.23 | 0.220 | 0.14 | 1.64 | 0.204 |
| Productive | 3.13 | 0.41 | 5.32 | **< 0.001** | 0.03 | 0.44 | 0.664 | 0.22 | 2.61 | **0.019** |
| Well | 3.05 | 0.65 | 7.12 | **< 0.001** | 0.16 | 1.64 | 0.205 | 0.02 | 0.19 | 0.850 |

Table C: Internal consistencies for long term measures at baseline and post study.

| **Subscale** | **Baseline ωt** | **Post Study ωt** |
| --- | --- | --- |
| DASS Depression | 0.92 | 0.93 |
| DASS Anxiety | 0.86 | 0.84 |
| DASS Stress | 0.88 | 0.85 |
| MWQ | 0.88 | 0.91 |
| QOLI | 0.84 | 0.80 |
| MAAS | 0.91 | 0.89 |
| HMS | 0.96 | 0.96 |
| M5 Extraversion | 0.90 | 0.91 |
| M5 Agreeableness | 0.85 | 0.84 |
| M5 Conscientiousness | 0.85 | 0.83 |
| M5 Neuroticism | 0.93 | 0.91 |
| M5 Openness | 0.80 | 0.82 |
| TAS | 0.89 | 0.90 |
| SOARS Involuntariness | 0.78 | 0.75 |
| SOARS Effortlessness | 0.85 | 0.81 |

Table D: Full linear effects model summary for long term measures in Study One. This table shows uncorrected p-values. The p-values in the manuscript are corrected using the Holm-Bonferroni adjustment for the three critical tests (main effect of time, interaction of time x experience, and interaction of time x dose).

|  |  | **Time** | | | **Experience** | | | **Doses** | | | **Time x Experience** | | | **Time x Doses** | | |
| --- | --- | --- | --- | --- | --- | --- | --- | --- | --- | --- | --- | --- | --- | --- | --- | --- |
|  | **Intercept** | ***B*** | ***t*** | ***p*** | ***b*** | ***t*** | ***p*** | ***b*** | ***t*** | ***p*** | ***b*** | ***t*** | ***P*** | ***b*** | ***t*** | ***p*** |
| **DASS_Depression** | 4.81 | 1.24 | 3.97 | < 0.001 | 0.90 | 1.87 | 0.067 | -0.02 | -0.23 | 0.821 | 0.17 | 0.53 | 0.596 | -0.02 | -0.44 | 0.660 |
| **DASS_Anxiety** | 3.10 | 0.45 | 1.95 | 0.056 | 0.78 | 2.20 | 0.032 | 0.01 | 0.20 | 0.843 | -0.08 | -0.36 | 0.723 | -0.01 | -0.20 | 0.842 |
| **DASS_Stress** | 6.25 | 0.99 | 3.36 | 0.001 | 0.96 | 2.21 | 0.031 | -0.02 | -0.28 | 0.777 | -0.03 | -0.11 | 0.912 | -0.03 | -0.67 | 0.507 |
| **MWQ_Mean** | 3.69 | 0.15 | 2.49 | 0.016 | 0.05 | 0.41 | 0.682 | 0.01 | 0.50 | 0.616 | 0.00 | 0.06 | 0.956 | 0.00 | -0.19 | 0.853 |
| **QOLI_Total** | 1.78 | -0.13 | -1.41 | 0.165 | -0.30 | -1.49 | 0.141 | 0.00 | 0.10 | 0.919 | 0.07 | 0.73 | 0.469 | 0.00 | 0.28 | 0.783 |
| **MAAS_Mean** | 3.84 | -0.07 | -1.32 | 0.191 | -0.02 | -0.22 | 0.823 | 0.01 | 0.42 | 0.679 | -0.01 | -0.13 | 0.900 | -0.01 | -2.00 | 0.051 |
| **HMS_Total** | 119.80 | 0.48 | 1.08 | 0.283 | -2.59 | -3.67 | 0.001 | 0.05 | 0.49 | 0.629 | -0.21 | -0.46 | 0.645 | 0.00 | -0.01 | 0.990 |
| **M5_Extraversion** | 3.06 | 0.02 | 1.03 | 0.309 | -0.01 | -0.20 | 0.841 | 0.00 | 0.51 | 0.612 | 0.00 | 0.09 | 0.928 | 0.00 | 1.22 | 0.229 |
| **M5_Agreeableness** | 3.28 | -0.04 | -1.97 | 0.054 | -0.03 | -0.75 | 0.456 | 0.01 | 1.85 | 0.069 | -0.02 | -1.02 | 0.31 | 0.00 | -1.26 | 0.211 |
| **M5_Conscientiousness** | 2.64 | -0.02 | -1.25 | 0.215 | 0.04 | 1.24 | 0.220 | 0.00 | 0.87 | 0.386 | -0.04 | -2.38 | 0.021 | 0.00 | -1.41 | 0.163 |
| **M5_Neuroticism** | 3.21 | -0.05 | -2.70 | 0.009 | -0.06 | -1.07 | 0.289 | -0.01 | -0.57 | 0.568 | -0.03 | -1.86 | 0.068 | 0.00 | -0.46 | 0.647 |
| **M5_Openess** | 2.68 | -0.01 | -0.31 | 0.757 | -0.01 | -0.39 | 0.701 | 0.00 | 0.31 | 0.757 | -0.01 | -0.49 | 0.625 | 0.00 | -1.16 | 0.249 |
| **TAS_Total** | 21.78 | -1.11 | -4.46 | < 0.001 | -1.34 | -1.55 | 0.126 | 0.06 | 0.50 | 0.617 | -0.31 | -1.26 | 0.214 | 0.03 | 0.84 | 0.402 |
| **CPS_Total** | 61.40 | 0.13 | 0.55 | 0.588 | -0.72 | -1.91 | 0.062 | -0.03 | -0.56 | 0.578 | 0.35 | 1.40 | 0.166 | -0.03 | -0.70 | 0.489 |
| **SOARS_Involuntariness** | 19.43 | 0.50 | 2.18 | 0.033 | 0.25 | 0.78 | 0.437 | -0.02 | -0.51 | 0.615 | 0.12 | 0.52 | 0.608 | 0.02 | 0.56 | 0.577 |
| **SOARS_Effortlessness** | 20.31 | -0.61 | -2.31 | 0.024 | -0.27 | -0.77 | 0.446 | 0.05 | 1.01 | 0.315 | 0.09 | 0.32 | 0.748 | 0.04 | 0.91 | 0.367 |

Table E: Mean weighted expectation scores and ranks for naïve and experienced participants in Study Two.

| **Variable** | **Naïve Weighted Expectation** | **Naïve Rank** | **Experienced Weighted Expectation** | **Experienced Rank** |
| --- | --- | --- | --- | --- |
| CPS total score | 0.95 | 1 | 0.92 | 2 |
| QOLI total score | 0.92 | 2 | 0.92 | 3 |
| Mean MAAS score | 0.91 | 3 | 0.93 | 1 |
| Openness | 0.86 | 4 | 0.89 | 4 |
| HMS total score | 0.84 | 5 | 0.89 | 5 |
| Conscientiousness | 0.76 | 6 | 0.69 | 8 |
| Agreeableness | 0.72 | 7 | 0.81 | 6 |
| Extraversion | 0.70 | 8 | 0.81 | 7 |
| TAS total score | 0.64 | 9 | 0.69 | 9 |
| Effortlessness | 0.39 | 10 | 0.55 | 10 |
| Involuntariness | -0.11 | 11 | -0.09 | 11 |
| Mean MWQ score | -0.29 | 12 | -0.13 | 12 |
| Neuroticism | -0.78 | 13 | -0.64 | 13 |
| Anxiety | -0.80 | 14 | -0.71 | 14 |
| Stress | -0.85 | 15 | -0.83 | 15 |
| Depression | -0.87 | 16 | -0.89 | 16 |
